# Prevalence and patterns of dietary supplement use in elite Spanish athletes

**DOI:** 10.1186/s12970-019-0296-5

**Published:** 2019-07-18

**Authors:** Gabriel Baltazar-Martins, Diego Brito de Souza, Millán Aguilar-Navarro, Jesús Muñoz-Guerra, María del Mar Plata, Juan Del Coso

**Affiliations:** 1 Exercise Physiology Laboratory, Camilo José Cela University, C/ Castillo de Alarcón, 49. Villafranca del Castillo, 28692 Madrid, Spain; 2 Exercise and Sport Sciences. Faculty of Health Sciences, Francisco de Vitoria University, Madrid, Spain; 3 Department for Doping Control, Spanish Agency for Health Protection in Sport, Madrid, Spain; 4 Department of Education, Spanish Agency for Health Protection in Sport, Madrid, Spain

**Keywords:** Dietary supplement, Ergogenic aid, Athletic performance, Elite athlete

## Abstract

**Background:**

Dietary supplementation is a common strategy to achieve a specific health status or performance benefit. Several investigations have focused on the prevalence of dietary supplement use by athletes. However, information on how athletes manage the use and purchase of dietary supplements is scarce.

**Methods:**

Five hundred and twenty-seven high-performance athletes (346 males and 181 females), participating in individual and team sports, completed a validated questionnaire about use and purchase patterns of dietary supplements. The dietary supplements were categorized according to the International Olympic Committee (IOC) consensus.

**Results:**

Sixty four percent of the athletes (*n* = 337) used dietary supplements (median = 3; range 1 to 12). Age, sex, type of sport, level of competition, and professionalism influenced the prevalence of dietary supplement use (all *p* < 0.05). The most prevalent dietary supplement consumed was proteins (41%; *n* = 137), followed by amino acids/BCAA-based supplements (37%; *n* = 124). Additionally, as per group of supplements according to IOC consensus, 18% of the supplements were rated as having a low level of scientific evidence (e.g., glutamine, HMB, L-carnitine, etc). Most athletes (45%, *n* = 152) purchased dietary supplements in a store and 24% (*n* = 81) obtained them from a sponsor. Most athletes also (42%, *n* = 141) reported a self-organization of supplementation and did not consult with any professional. Last, 81% (*n* = 273) of athletes consuming supplements did not know any platform to check supplement safety/quality. For those who do not use dietary supplements (36% of the total sample, *n* = 190), most reported that they do not consider supplements necessary (72%, *n* = 137).

**Conclusion:**

Dietary supplementation appears to be widely used in sport with a considerable proportion of athletes consuming supplements with low level of scientific evidence. Additionally, athletes seem to rely on inadequate sources of information and may be largely unaware of sources to detect supplement contamination.

## Background

The quest for optimal nutrition has been gaining importance among athletes as the level of sport competition has become more demanding [1]. At the elite level, the constant quest for excellence is obtained through regular and planned training, while the advances in nutrition and supplementation can aid to improve athlete’s performance, recovery, health and well-being [2].

A dietary supplement is a commercially available product that is ingested in addition to the habitual diet. Athletes often use dietary supplements as a strategy to achieve a specific health outcome or exercise performance benefit [3, 4]. Although some consider that the use of nutritional supplements is unnecessary when athletes have a well-balanced diet [5], dietary supplement use has grown significantly in the past years [6]. Dietary supplements are available for the general population, but the use of these supplements is higher in elite athletes than in non-athletes or recreationally active individuals [3]. The overemphasis of dietary supplement use, as endorsed by internet and social media, along with the efforts of nutritional supplement companies to sponsor remarkable athletes [7] have aided at increasing the use of these products worldwide. In 2017, global sales of supplements reached US $128 billion [8]. Although the use of supplements varies across different sports, its usage is generally higher in men than in women and increases with age [4]. In addition, the athletes involved in short sprint-based activities typically consume less dietary supplements than athletes competing in endurance-based activities [9].

The prevalence of dietary supplement use by athletes has been the topic of several investigations [10, 11] and their results have been recently reviewed and systematically analyzed [4]. Overall, the prevalence of supplement consumption ranges from approximately 48 to 81% [12–17] while proteins and multivitamins are the most popular supplements. The reasons reported by athletes for using dietary supplements are diverse although are mainly related to health-related issues [16], physical and mental performance improvement [18], and increased rate of recovery [1]. However, the patterns of use and purchase of supplements have not been well investigated.

Athletes often rely on their coaches [15], family [16], and friends/teammates [19] as their preferable sources of reliable information for their use of dietary supplements. However, sports nutritionists or scientists are rarely the main source of information to plan a supplementation program [20], even at the elite level. This may lead athletes to an excessive and/or incorrect use of dietary supplements along with possible adverse interactions due to polypharmacy [21]. In addition, there are significant risks associated with the use of dietary supplements, such as the absence of active ingredients, the presence of harmful substances, or even the presence of doping agents [22]. With rates of contamination between 12 and 58% [23], the likelihood of unintended doping with the use of supplements is high. Lastly, athletes are not always aware of the risks associated with purchasing supplements and rarely inform themselves to confirm the quality and safety of the selected supplement [20]. The internet has become a preferable site to purchase supplements and thus, the easy access to contaminated nutritional supplements and “black market” products might constitute a risk for public health.

Since athletes often use dietary supplements with no clear understanding of their effects and risks [24], it becomes critical to provide information about the patterns of use and purchase of dietary supplements in samples of elite athletes. This information might help to provide nutritional education approaches that reduce the risk associated with dietary and nutritional supplementation through better informed the athletes. Taking into account the lack of research in Spanish athletes [25, 26], the present study aimed to determine the prevalence of supplement use by elite athletes while describing how athletes manage dietary supplements use.

## Methods

### Participants

Five hundred and twenty-seven athletes (346 males and 181 females) volunteered to participate in this investigation by filling out a validated and standardized questionnaire about the use of dietary supplements [27]. The athletes were considered elite because all of them were training and competing in high-performance programs of different national sports federations. Specific information about the study sample can be obtained in Table 1. The questionnaire was provided by email to athletes with the help of staff from different national federations and announcements in sport performance centers. Thus, it was unfeasible to record the number of athletes solicited for this investigation. Forty-five athletes were excluded from the study because they did not complete the questionnaire and 4 questionnaires were not considered valid because they contained duplicate information in all questions. Informed consent was obtained with the questionnaire. The study was approved by the Camilo José Cela University Ethics Committee and it was carried out in accordance with the procedures approved by the Declaration of Helsinki.

**Table 1:** Socio-demographic characteristics of the participants and distribution of athletes who reported use/not use of supplements in the last year

	Total	Frequency % (n)		
		Yes	No	*P* value
Gender				
Male*	346	67% (232)	33% (114)	0.04
Female*	181	58% (105)	42% (76)	
Total	527	64% (337)	36% (190)	
Age Range				
< 15–20 years*	111	30% (33)	70% (78)	< 0.01
21–25 years	123	65% (80)	35% (43)	
26–30 years*	106	77% (82)	23% (24)	
31–35 years	58	74% (43)	26% (15)	
36–40 years*	62	79% (49)	21% (13)	
> 41 years	66	74% (49)	26% (17)	
Sport				
Body Building*	38	95% (36)	5% (2)	< 0.01
Cycling	36	86% (31)	14% (5)	
Athletics	238	77% (184)	23% (54)	
Triathlon	75	77% (58)	23% (17)	
Aquatics	31	77% (24)	23% (7)	
Weightlifting	16	75% (12)	25% (4)	
Football	8	75% (6)	25% (2)	
Volleyball	8	75% (6)	25% (2)	
Others	112	74% (83)	26% (29)	
Canoe/Kayaking	11	73% (8)	27% (3)	
Field Hockey	11	64% (7)	36% (4)	
American Football*	68	57% (39)	43% (29)	
Golf*	29	55% (16)	45% (13)	
Gymnastics*	17	53% (9)	47% (8)	
Basque pelota (jai alai)*	15	53% (8)	47% (7)	
Level of competition				
National*	262	71% (262)	29% (77)	< 0.01
International*	265	57% (265)	43% (113)	
Professional				
Yes*	85	75% (64)	25% (21)	0.02
No*	438	61% (269)	39% (169)	

(*)The distribution was different from the value expected at *p* < 0.05

### Questionnaire

The questionnaire used in this investigation has been previously validated to assess the prevalence of dietary supplement use and to obtain information about the individual consumption patterns in the last year [27]. The questionnaire also assessed information on socio-demographic variables, sport level, and professionalism. This questionnaire was developed by a group of experienced sport scientists, its construct validity was verified by a group of six experts in nutrition, sports sciences and chemistry (Aiken’s V = 0.97 for pertinence and 0.82 for relevance of the questions) and its reliability has been measured by a test-retest performed 4 weeks apart. The questionnaire contained a definition of a dietary supplement according to the latest consensus statement of the International Olympic Committee (IOC; [3]). Through 81 questions, it also asked participants about the use of performance enhancing substances, supplements for weight control, supplements to increase the rate of recovery, medicaments, and other substances. The questionnaire allowed athletes to report the total number, type of supplement used, and season of consumption (pre-season, competitive periods, or all year). The questionnaire also had a section to be filled out only by those who did not report any supplement use in order to ascertain the reasons for their lack of use.

The questionnaire was organized to obtain information about a) sociodemographic information, sport discipline, and level of competition; b) prevalence and frequency of dietary supplement usage; c) reasons for the use of supplements, sources of information about supplementation, and contamination and purchase conditions. Participants filled out the questionnaire between July 2017 and May 2018. In order to help athletes to identify supplements, examples for each category were provided.

### Analysis of dietary supplements by group

To improve the applicability of the results, each supplement was individually notated and grouped according to the groups of the IOC consensus statement [3], as follows:

1. “Performance enhancement”, which included caffeine, beta alanine, creatine, sodium bicarbonate, carbohydrate foods, and carbohydrate powders.
2. “Immune health”, which included antioxidant supplements, probiotics, and vitamin C.
3. “Micronutrients”, which included iron supplements, magnesium, folic acid, calcium, zinc, selenium, multivitamin supplements, and electrolytes.
4. “Improve recovery & injury management”, which includes joint support supplements (glucosamine, chondroitin, collagen), recovery supplements (mixes of carbohydrate and protein powders labelled as a “recovery product”), omega- 3 &- 6 polyunsaturated fatty acids, and curcumin.
5. “Body composition changes”, which includes protein powders (whey protein mixes, casein, calcium caseinate, plant/meat/egg-based protein powders).
6. “Low level of evidence supplements”, which includes: glutamine, single amino acids/branched-chain amino acids (BCAA), beta-hydroxy beta-methylbutyrate (HMB), L-carnitine, spirulina, royal jelly, citrulline, probiotics, taurine, conjugated linoleic acid, co-enzyme Q10, and fat burners, among others.

It is important to clarify that some dietary supplements may be included in different categories, as reported in the IOC consensus [3]. However, we have selected the most pertinent category based on scientific evidence. This is the case of “carbohydrate powders” (including maltodextrin, amylopectin, and powdered isotonic blends) and “carbohydrate foods” (energy bars, energy gels, and other miscellaneous carbohydrate-rich products) which have been included in the “performance enhancement” category. Although the IOC consensus also considers them as supplements for immune health, the evidence so far mainly supports their role in the maintenance of exercise intensity in endurance sports [28]. Similarly, other dietary supplements reported to have a low level of evidence have been included in a category of their own (“low level of evidence supplements”). Although some of these supplements may be in a specific category in the IOC consensus statement, these are reported in said document has having a low level of evidence (e.g. glutamine and HMB). Finally, athletes from 23 different sport disciplines filled out the questionnaire, but sport disciplines with less than 8 participants were grouped in the “others” category.

### Statistical analysis

After the data collection, data was organized, checked and analysed with the statistical package SPSS 20 (SPSS Inc., Chicago, IL). Participants and quantitative data of dietary supplements are expressed by frequencies and percentages. Because the number of supplements used followed a non-normal distribution, median and range have been calculated for this variable. The 16 most-used supplements have been presented for clarity. The differences in the distribution of dietary supplements use across the groups made by sociodemographic variables (Table 1) were tested with crosstabs and the Chi Square test, including adjusted standardized residuals. The significance level was set at *p* < 0.05.

## Results

From the total sample, 64% of the athletes (*n* = 337) reported habitual use of at least one dietary supplement in the last year during any point of the season. The remaining 36% of the sample (*n* = 190) did not report any supplement use in the last year. Overall, male athletes reported a higher use of dietary supplements than women (*p* = 0.04; Table 1) while age was another variable that significantly modified the prevalence of use (*p* < 0.01). Although all sports showed a supplement usage frequency of at least 50%, body building was the discipline with the highest self-reported use of supplements (Table 1). Cycling, athletics, triathlon, and aquatics were also sport disciplines with high proportions of athletes using supplements (Table 1). American football, golf, gymnastics, and Basque pelota had lower than expected frequencies in the proportion of athletes that used supplements (*p* < 0.01; Table 1). The use of supplements was higher in athletes that had national-level standings than athletes with international-level standings (*p* < 0.01) while professionalism increased the use of supplements (*p* = 0.02).

**Fig. 1:**
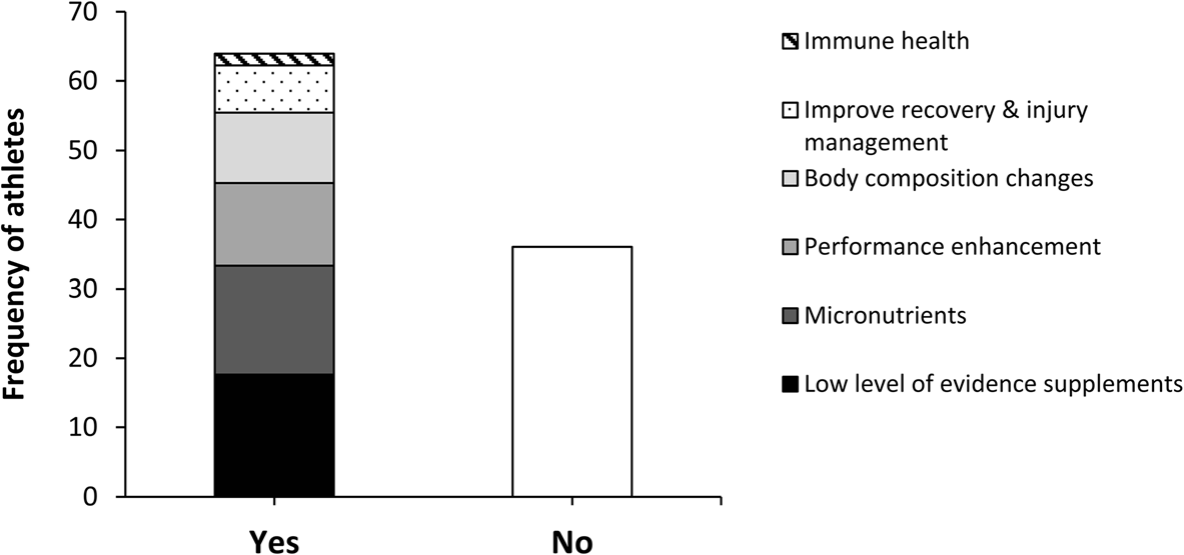
Distribution of dietary supplement use in elite athletes according to the categories used in the International Olympic Committee consensus statement on dietary supplements and the high-performance athlete

**Fig. 2:**
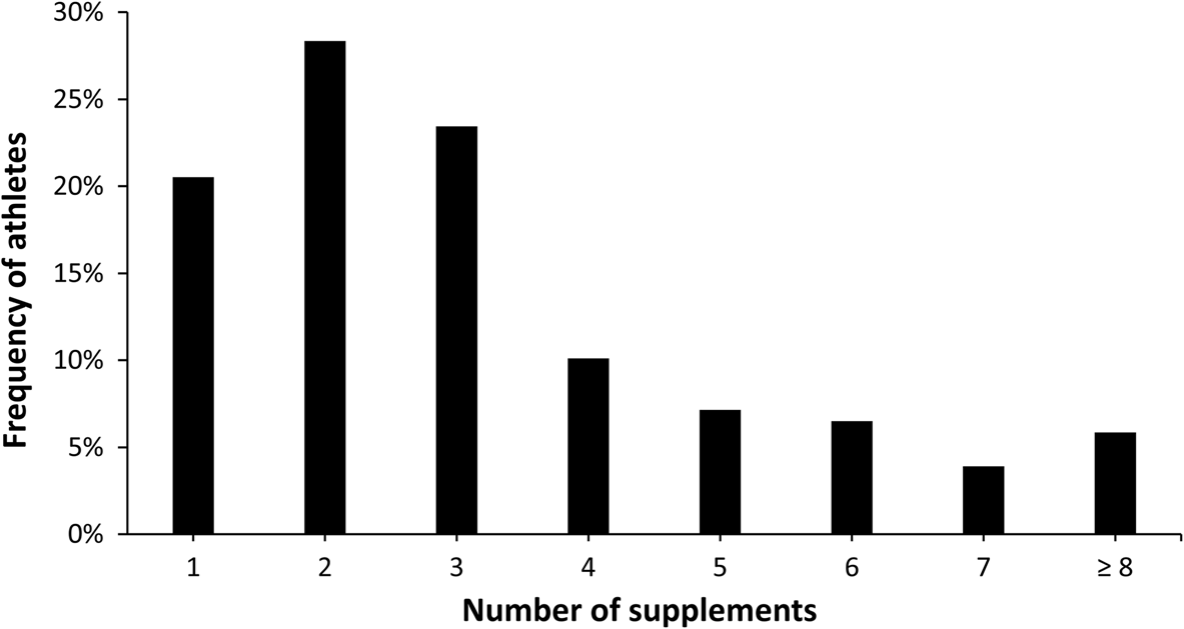
Frequency of elite athletes according to the number of supplements used in the last year

**Fig. 3:**
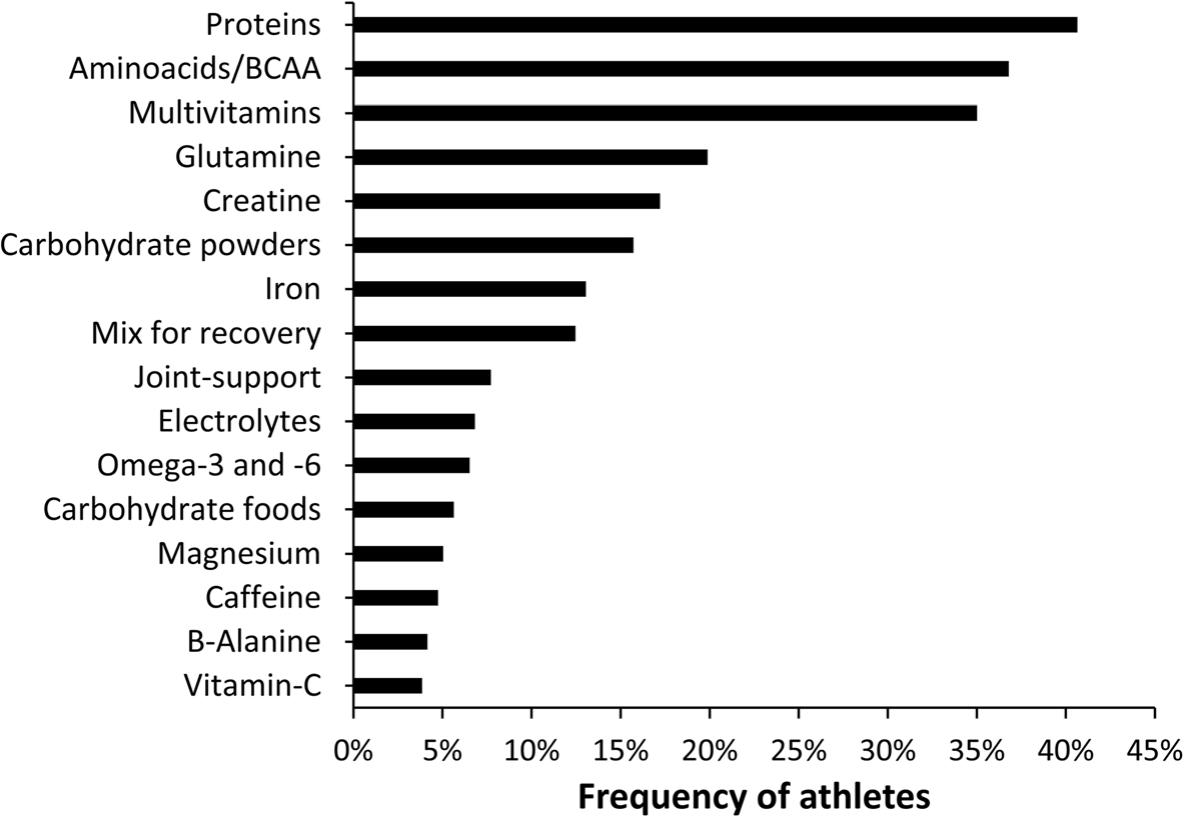
Frequency of elite athletes using the 16 most taken supplements in the last year

**Fig. 4:**
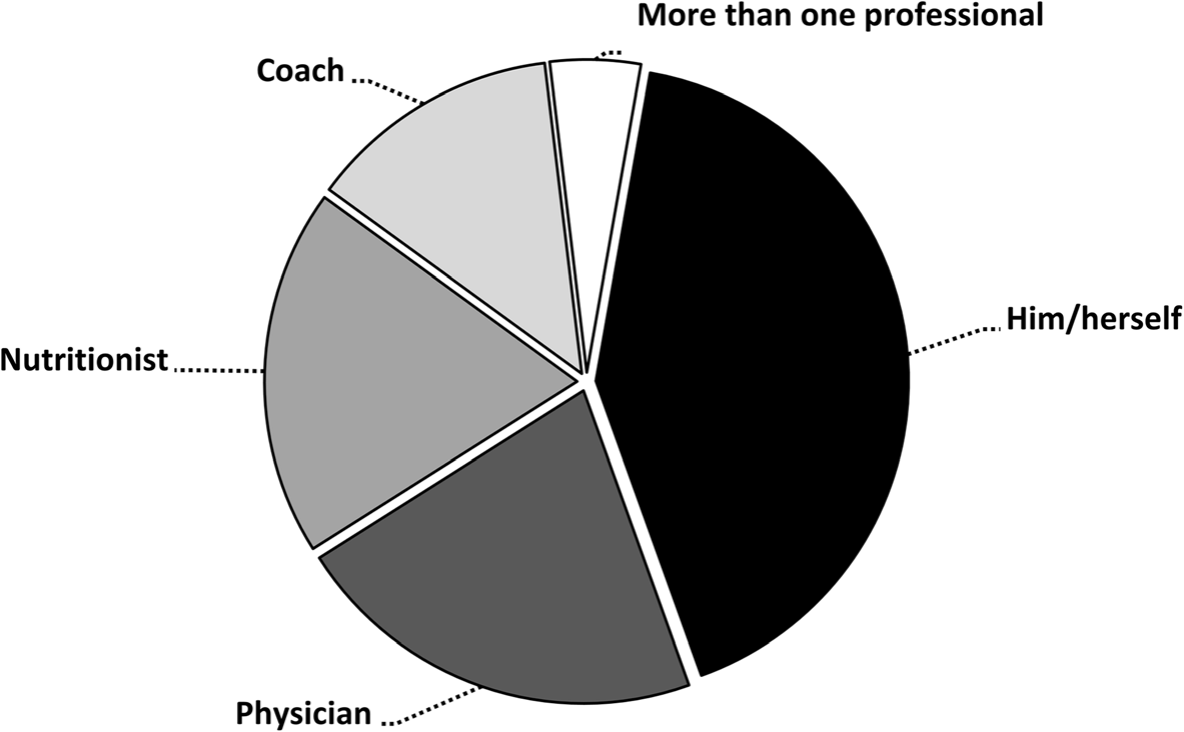
Main source of information to determine the type, use, and utility of dietary supplementation in elite athletes

**Fig. 5:**
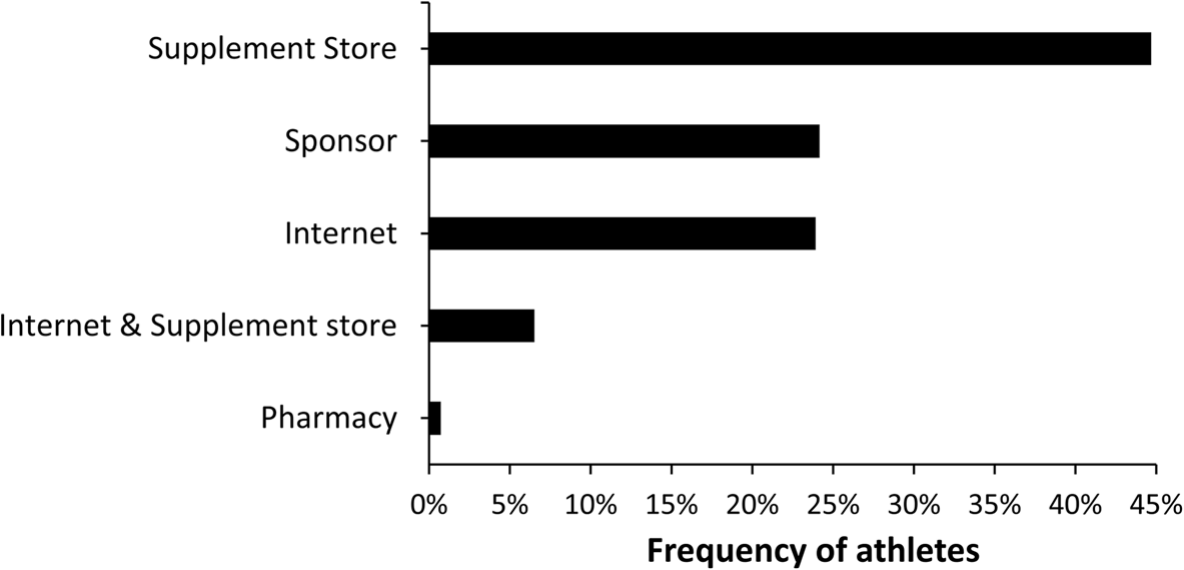
Main site of dietary supplements purchases in elite athletes

In total, 1056 supplements were reported; most of the supplements were categorized as low level of evidence substances, followed by micronutrients and performance enhancement supplements (Fig. 1). In the sample of supplements users, a median consumption of 3 supplements per athlete was found with a range from 1 to 12 supplements (Fig. 2). Still, 6% of athletes reported a use of ≥8 supplements in the last year. From the subsample of supplement users, 47% reported consumption during the whole season, 43% reported consumption only during competitive periods, and 10% reported consumption only during the pre-season. Proteins, amino acids/BCAA, and multivitamins were the most consumed supplements in the sample of supplement-users (Fig. 3). A total of 36 different supplements were identified in the questionnaire. Most of the athletes that consumed supplements reported relying on him/herself for the obtaining of valid and accurate information about the efficacy of the supplements and they did not consult any professional for this matter (Fig. 4). The remaining athletes reported seeking advice through physicians, nutritionists, and coaches as alternative sources of information. The most common site of purchase were physical supplement stores while a high proportion of athletes directly obtained supplements from sponsors or internet websites (Fig. 5). Although 85% of the sample indicates that they only used certified supplements free of doping agents, 81% were not aware of any platform to verify supplement safety/quality. This is because 92% of the sample considered that supplements are safe and controlled by the supplement company/brand. Only 40% of athletes had knowledge of a Spanish national-based application that certified permitted supplements and identified doping agents (i.e., NoDop App).

**Fig. 6:**
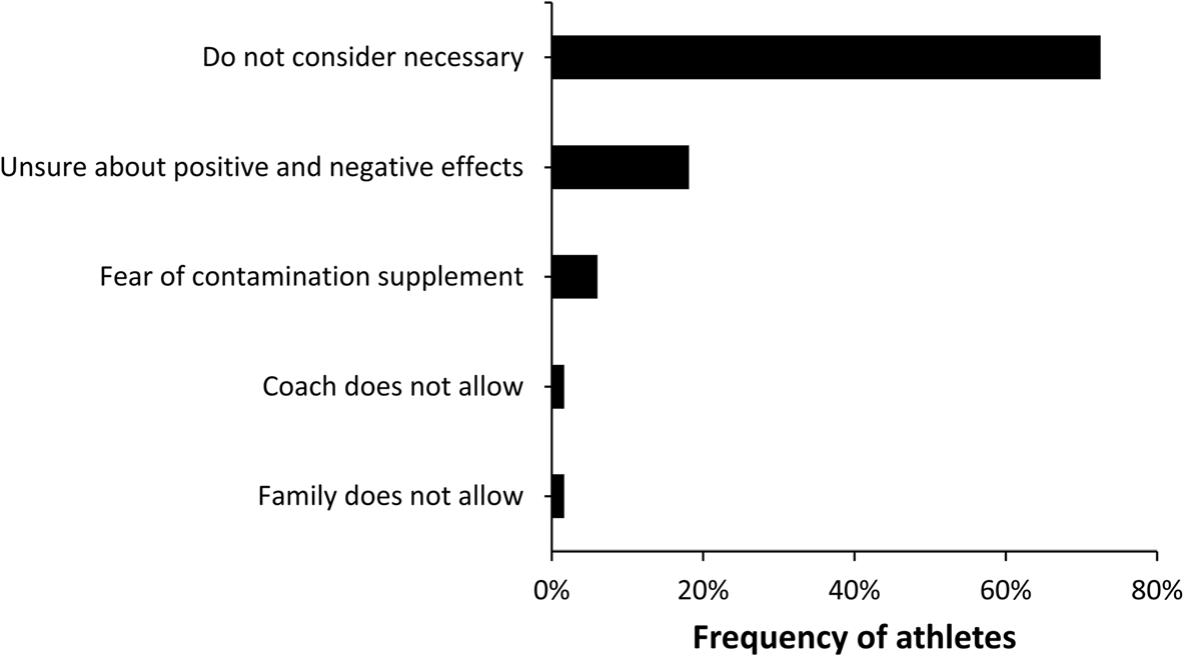
Relative frequency of athletes not taking supplements according to the reasons for not using them

Among the athletes that did not report any use of supplements (*n* = 190), the main reason was that they did not consider them necessary to maintain their level of fitness (Fig. 6). A low proportion of athletes did not consume supplements because their family/coach did not allow this practice.

## Discussion

The aim of this study was to investigate the prevalence of dietary supplement usage in elite athletes from different sports and to provide information about how athletes manage the use of these supplements. We gathered information about the number and type of supplements consumed in the last year, when and why the supplements were consumed, along with data about purchasing routines and certification of supplement quality/safety. The current investigation was shaped by the recent suggestions for improving research on dietary supplement use as raised by Knapik et al. [4]. The investigation relied on a questionnaire that included definitions and examples of dietary supplements. It also contained specific categories of dietary supplements along with open and closed questions for participants to respond to. A wide variety of sports were solicited for questioning. Although a similar aim has been pursued in several previous investigations [12–16], this current study is novel because it expands upon the most common practices employed for the management and administration of dietary supplements in a sample of elite athletes. Finally, the study is innovative because categorizes dietary supplements according to the last consensus of the IOC [3].

Overall, the use of at least one dietary supplement was reported by 64% of the study sample, a proportion within the range of previous investigations carried out in similar samples in Europe [1, 14]. However, several demographic variables affected the proportion of athletes that consumed dietary supplements in the last year. Sex, age, level of competition, and professionalism influenced this proportion. The sociotype of a typical dietary supplement user is a 36–40-year-old male that competes at a national level, but in a sport that allows professionalism. Interestingly, age was the strongest predictor of dietary supplement use which confirms this variable as an important modulator of the decision to use supplements to obtain performance and/or health benefits, as previously found [13, 29]. The use of dietary supplements by youngest athletes is likely monitored by parents and coaches compared with older athletes who might have more opportunity to engage in unsupervised supplements use [30].

The sport discipline was another variable with great influence in the proportion of dietary supplements use (Table 1). The sport with the highest proportion of dietary supplement use was bodybuilding, with 95% of body-builders reporting the use of at least one supplement/year. Cycling, athletics triathlon, and aquatics also had high frequencies of dietary supplement use, as observed in elite Portuguese athletes, where the number of training hours –normally higher in endurance-based activities-- was associated with an increased supplement consumption [1]. Additionally, other studies also confirms that endurance athletes appear to consume more supplements than athletes engaged in sprint-based activities [9]. Interestingly, team sports (i.e., football, volleyball, field hockey, etc) presented a non-significant lower proportion of athletes that used supplements. This data coincides with previous publications in samples of Spanish tennis [25] and basketball players [12] where individual athletes reported a higher use of supplements than in team sports (81% vs 58%). Even in the sports with the lowest proportion of supplement use (i.e., gymnastics and Basque pelota), one out of two athletes reported the supplement use. This data reinforces the idea that characteristics of the sport influence the general use of dietary supplements in sports while it supports the establishment of dietary supplementation as a common tool for athletes of all types of sport disciplines.

The number of consumed supplements also presented a high interindividual variability with athletes consuming between 1 to 12 dietary supplements, as previously found [1, 16] Although the median of supplement consumption per athlete was 3 supplements per year, a high proportion of athletes consumed more than 8 different supplements and at different times of the season. This results suggest that some athletes might be subject to the adverse interactions of polypharmacy [21] while also laying out the idea of excessive dietary supplement use amongst some athletes. This is more evident by the high reliance of athletes on themselves as being the main source to obtain accurate information about the effect and efficacy of the supplement while they referred themselves as a the main responsible for the plan of supplementation (Fig. 4). As it has been found, receiving dietary counseling by a qualified professional - instead of relying on self-prescription - results in better-informed choices with respect to the use of nutritional supplements related to performance, recovery, and health [31]. This information points towards the necessity of increasing the knowledge of the benefits and risks of supplementation in the elite athlete population. This result highlights the importance of elite athletes placing more reliance on sport nutritionists and scientists to design their supplementation plans. A more informed athlete population will likely reduce the strong effect of purchasing multiple types of supplements that have been driven by dietary supplement manufacturers.

The most consumed dietary supplements were proteins, amino acids/BCAA, multivitamins, glutamine, and creatine (Fig. 3). A very similar pattern for the type of supplements consumed has been reported in other studies where proteins [32], multivitamins [31], and creatine [29] were found to be the most consumed substances. However, as a novelty of this investigation, the data indicate that the most prevalent group of substances were ones that had little scientific evidence (Fig. 1). This effect was produced by the high number of supplements available in the market that contain minimal evidence of its effectiveness. Although this in the first investigation that suggests this finding, it could be ventured from previous literature due to the gaps in knowledge about effective nutrition and supplementation found in coaches and athletes [24]. This outcome is likely the result of current supplementation practices that imply a poor knowledge about the effect and efficacy of supplements along with reliance on sources with low credibility, at least in this sample of high-performance athletes.

The internet is not only a readily accessible outlet for quick purchases, but also a source of information for the claimed effects of dietary supplements. It is also often reported as a preference site of purchase [33], as it was found in the present study. Nevertheless, the purchase of supplements in physical stores was listed as the main preference for athletes in this study. This might be justified by a possibly increased safety perception by athletes when buying supplements directly to the vendor when compared to buying online, but research on this topic is scarce and the justification of purchase preference merits further research. Because of their graphical attractiveness and ability to interact/share, athletes often prefer internet and social media as preferred tools for obtaining information and/or educating themselves on nutrition-related topics [29]. Possibly, an excessive amount of information readily available on the internet and a high engagement with social media [34] with marketing campaigns aimed for sports practitioners, might increase the risk for athletes not relying on other professionals to obtain advice.

Lack of legislation on dietary supplements worldwide, the risk of contamination, and the absence of proper information regarding their use and scientific basis [23, 24] may increase the risk for inadequate and excessive use of dietary supplements or even inadvertent doping.

In the sample of athletes that took at least one supplement, four out of five athletes did not know platforms to check safety/quality of supplements. Most solely relied on the brand name for quality and safety. Despite ample evidence that confirms contamination in commercially available products, athletes still purchased supplements with the assumption of safety [23]. Furthermore, only a relatively small percentage of the athletes not taking dietary supplements reported fearing contamination of the supplement. Together, all these results may indicate that athletes are disturbingly unaware of the contamination risks inherent in dietary supplements. Athlete supplement education is a critical need. This is important to not only reduce the cases of unintended doping [22], but to also avoid unintentional intakes of substances that could potentially have acute and long term side-effects [20].

This investigation presents some limitations that should be discussed to improve the results’ applicability. Although we used a validated and reliable questionnaire [27], the timeframe used to report the use of supplements (i.e., “in the last year”) might have induced some error due to imprecision in the number and type of supplements reported. This might be important for those subjects reporting a high number of supplements. We used open and closed questions and provided examples of each supplement category in an attempt to reduce recall inaccuracies. Additionally, although it was made clear that the questionnaire was anonymous, it is possible that due to personal bias, some athletes may have intentionally avoided reporting some information regarding supplement consumption. Finally, some athletes showed some difficulty describing the type of supplement they were taking. To avoid wrongfully identifying supplements, an open space was provided in the questionnaire to fully describe the supplement (name, brand, type, and any other extra information that they could recall) to improve the identification of each supplement. Despite these limitations, the authors believe that the article presents valuable information for the scientific community about patterns of dietary supplementation consumption.

## Conclusion

In conclusion, the results of this questionnaire demonstrate a widespread use of dietary supplements in elite athletes. Socio-demographic factors that include age, sex, type of sport, level of competition, and professionalism might influence the prevalence of dietary supplement consumption. Although proteins and amino acids were the most consumed supplements, substances with a low scientific basis for their consumption were the most predominant group of supplements. This is mainly due to the high number of commercially-available dietary supplements that fulfill this definition. Furthermore, athletes seem to rely on inadequate sources of information when acquiring and using supplements, with a considerable proportion of athletes engaging in self-prescription and purchase without consulting an accredited professional. Additionally, a high proportion of athletes are unaware of the contamination risks that dietary supplements may pose, which expose them to possible inadvertent doping. Urgent nutritional education and consulting should be made available to athletes and coaches, emphasizing the role of the nutritionist, sport scientists, and the acute and long-term side effects of incorrect plans of supplementation.

## Data Availability

Please contact author for data requests.

## Abbreviations

BCAA: Branched-chain amino acids
HBM: Beta-hydroxy beta-methylbutyrate
IOC: International Olympic Committee
